# Case Report: Use of reinforced buccal mucosa graft over gracilis muscle flap in management of post high intensity focused ultrasound (HIFU) rectourethral fistula

**DOI:** 10.12688/f1000research.10245.2

**Published:** 2017-02-13

**Authors:** Shrikant Jai, Arvind Ganpule, Abhishek Singh, Mohankumar Vijaykumar, Vinod Bopaiah, Ravindra Sabnis, Mahesh Desai

**Affiliations:** 1 Dept. of Urology, Muljibhaipatel Urological Hospital, Gujarat, India; 2 Staten Island University Hospital, Staten Island, USA

**Keywords:** HIFU, Urethro–rectal fistula, Fistula, Buccal Mucosa graft, Gracilis Muslce flap, Complicated Fistula, Carcinoma Prostate.

## Abstract

High intensity focused ultrasound (HIFU) has come forward as alternative treatment for carcinoma of the prostate. Though minimally invasive,HIFUhas potential side effects. Urethrorectal fistula is one such rare side effect. Management of these fistulas has been described by Vanni *et al. *

This case report describes points of technique that will help successful management of resilient rectourethral fistula. Urinary and faecal diversion in the form of suprapubic catheter and colostomy is vital. Adequate time between stoma formation, fistula closure and then finally stoma closure is needed. Lithotomy position and perineal approach gives best exposure to the fistula. The rectum should be dissected 2cm above the fistula; this aids in tension free closure of the rectal defect. Similarly buccal mucosal graft was used on the urethra to achieve tension free closure. A good vascular pedicle gracilis muscle flap is used to interpose between the two repairs. This not only provides a physical barrier but also provides a vascular bed for BMG uptake. Perfect haemostasis is essential, as any collection may become a site of infection thus compromising results.

We strongly recommend rectourethral fistula be directly repaired with gracilis muscle flap with reinforced buccal mucosa graft without attempting any less invasive repairs because the “first chance is the best chance”.

## Introduction

High intensity focused ultrasound (HIFU) is a treatment option in the management of prostate cancer ^1^. When combined with transurethral resection of prostate (TURP), risk of post procedure retention of urine and other side effects are significantly reduced. Urethrorectal fistula is a serious complication of HIFU. Literature reports a rate of urethrorectal fistula following HIFU ^2^ of approximately 0.7%. This case report describes management of recurrent urethrorectal fistula after HIFU with buccal mucosa graft (BMG) over a bed of gracilis flap.

## Case report

A 52-year-old man was evaluated for lower urinary tract symptoms (LUTS) and found to have raised PSA levels of 18.70 ng/ml. Transrectal ultrasound (TRUS) guided biopsy showed adenocarcinoma of the prostate with a Gleason’s score of 3+4 with evidence of extracapsular spread on the left side. Bone scan showed osteoblastic activity at the distal end of the right femur. Ultrasound (USG) showed 30 g prostate. He underwent an initial TURP to debulk the gland. Following the intervention in the same sitting he underwent HIFU. Histopathology showed 50% of the cores were positive for adenocarcinoma with a Gleason’s score of 4+4. The Foley catheter (PUC) was removed on the 5 ^th^ post-operative day (POD). On the 15 ^th^ POD, the patient had urine leak via the rectum. Diagnostic cystoscopy showed a single fistulous opening above the level of the external sphincter. As conservative management failed in form of suprapubic catheter (SPC), he underwent robotic assisted laparoscopic excision of the fistula. The bladder and rectum were closed separately with interposition of an a cellular matrix sheet in between.

**Figure 1.**
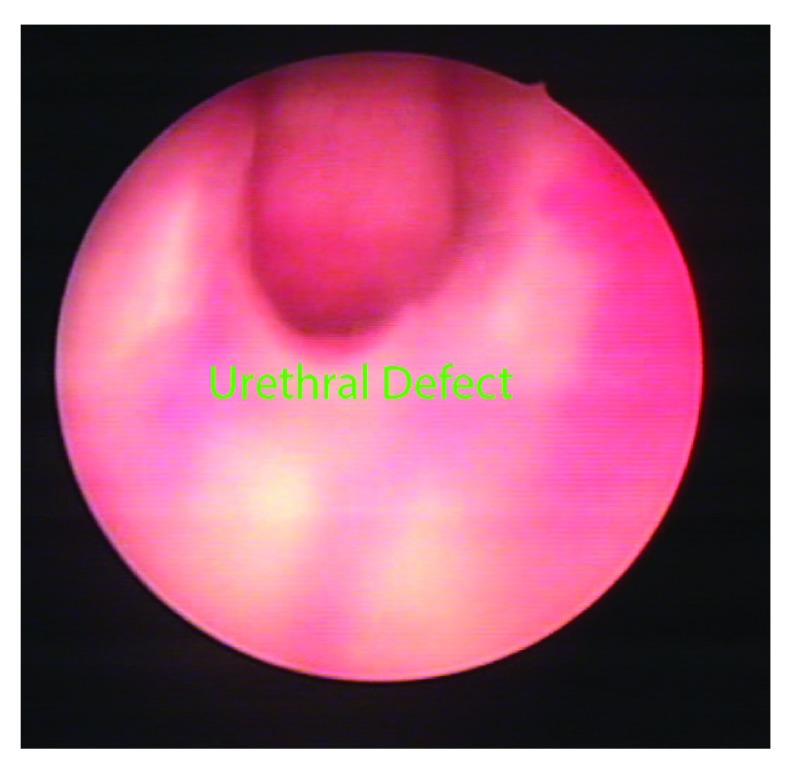
Cystouethroscopy showing urethral defect.

On the 6 ^th^ POD following robotic repair, the patient developed fecaluria which was managed with loop sigmoid colostomy and SPC. Repeat cystoscopy after 3 months showed persistent fistula ( Figure 1). He was planned for repeat surgery via perineal approach in view of his previous failed abdominal surgery and faecal contamination of abdomen.

**Figure 2.**
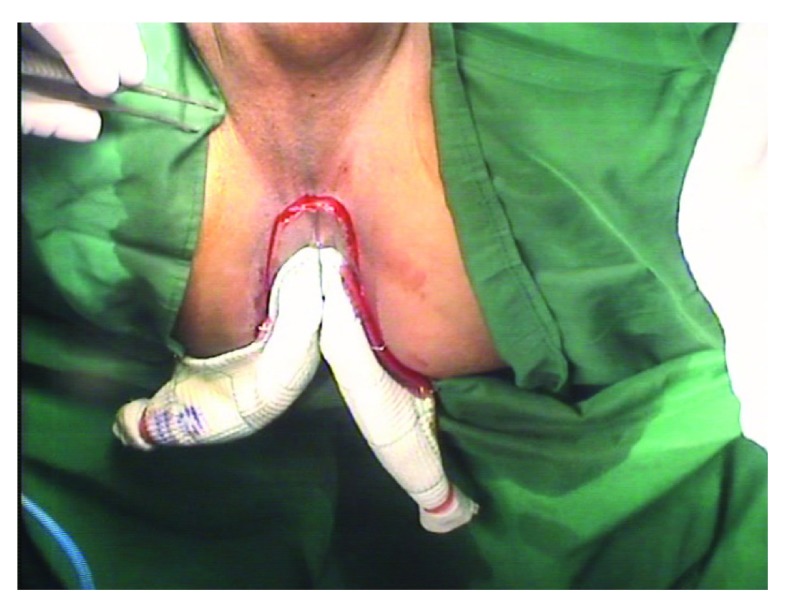
Perineal incision above anus.

**Figure 3.**
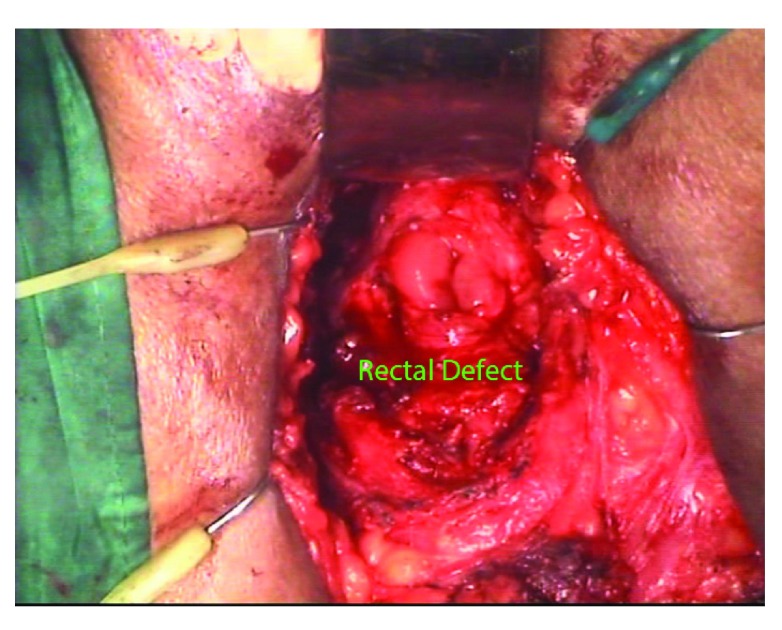
Rectal wall defect at the level of fistula.

**Figure 4.**
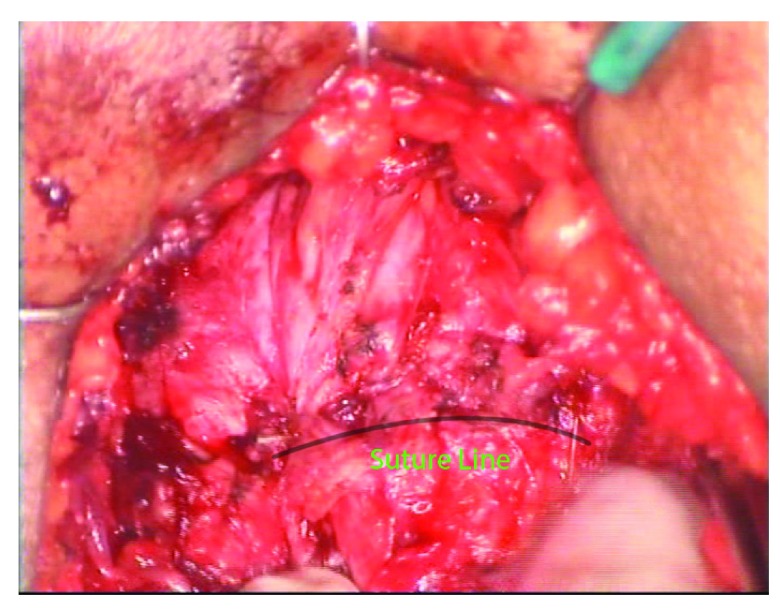
Suture line of rectal wall defect repair.

**Figure 5.**
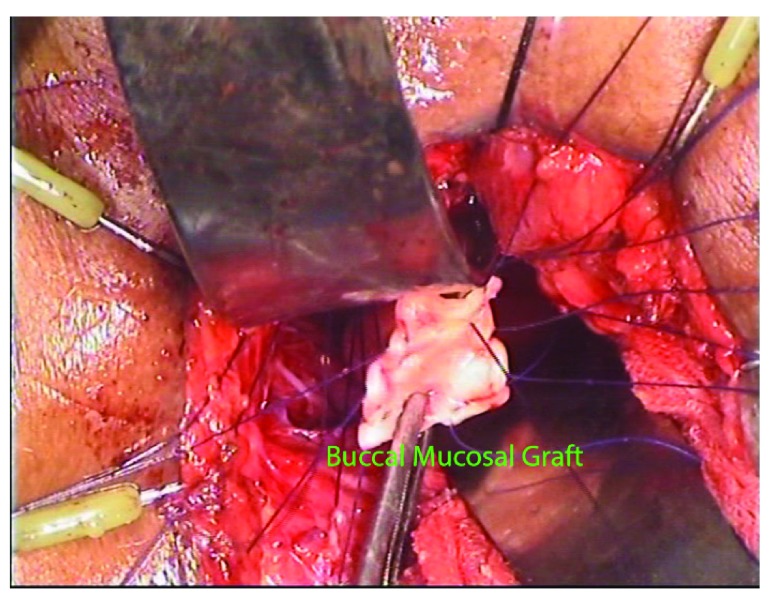
Buccal mucosal graft (BMG) with anchoring sutures.

**Figure 6.**
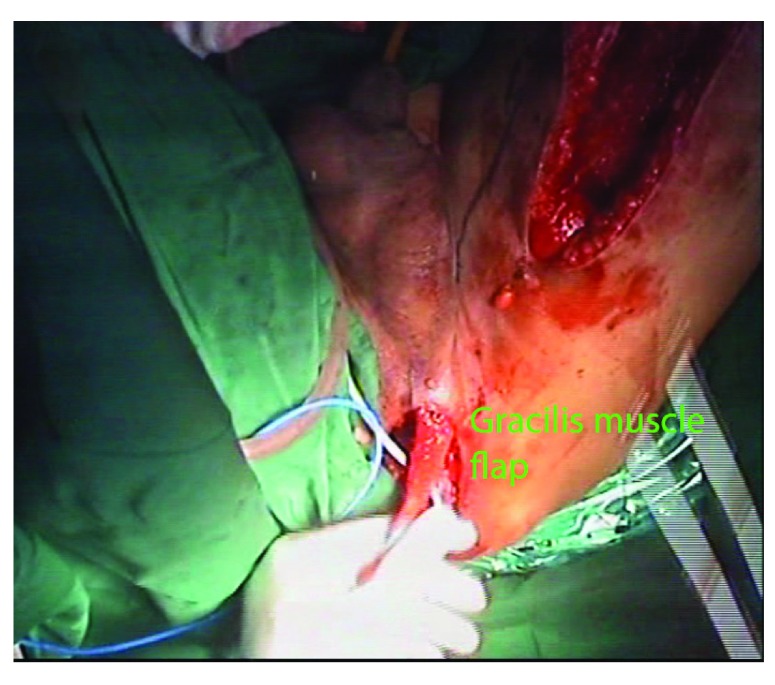
Gracilis muscle flap rotated to perineum.

**Figure 7.**
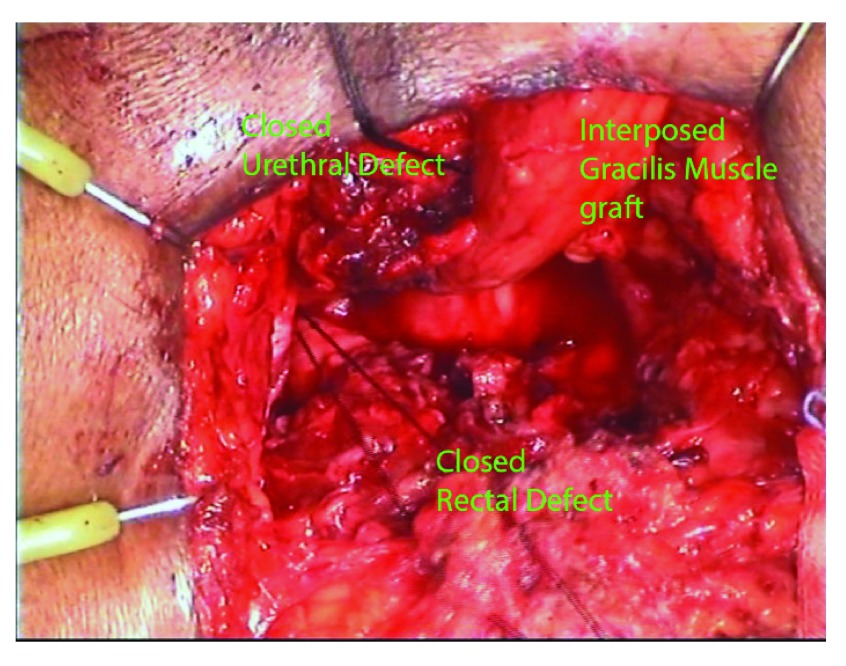
Gracilis muscle flap interposed and fixed between rectal and urethal repair.

The patient was placed in lithotomy position with the perineum nearly horizontal. An inverted smiling incision was made in the perineum above the anus ( Figure 2). Dissection showed dense adhesions between the rectum and surrounding tissue. Digital rectal examination done intraoperatively ensured rectal wall integrity. The fistula was at the 1 o’ clock position between the prostatic urethra and rectum ( Figure 3). All scar tissue and fistula was excised to create healthy margins. The rectal defect was repaired in transverse fashion in a single layer with monocryl 2-0 sutures ( Figure 4). BMG was harvested and positioned to bridge the urethral defect; it was anchored with interrupted 3-0 monofilament sutures ( Figure 5). A separate incision was made on the left thigh from the adductor tubercle to 2cm above the medial condyle. The gracilis muscle flap was harvested, rotated towards the perineum ( Figure 6) and interposed between the rectal and urethal repair ( Figure 7). Prior to closure of wound, adequate haemostasis was ensured.

On the 14 ^th^ POD the PUC was removed and SPC blocked. The patient was voiding well with a satisfactory uroflow without any leak of urine from the rectum. Colostomy closure was done after 3 months. On follow-up visits at 3 and 6 months, the patient was asymptomatic.

## Discussion

The grascilis muscle flap was first described by Ryan *et al* ^3^ for closure of rectouretral fistula. The gracilis muscle flap fulfills all the criteria of an ideal flap for interposition in such situations due to its rich vascular supply and ease of rotation.

Rabau *et al* ^4^ described a series of 10 patients who under went grascilis flap repair for rectourethral or rectovaginal fistula. Of these, 3 patients had fistula post radical prostatectomy and a prior failed attempt of fistula repair. On mean follow-up at 26 months they reported a 100% success rate. The results of our report closely resemble those of Michab *et al*.

In a series of 35 patients by Ulrich *et al* ^5^, 4 patients had fistula post radical prostatectomy and all were treated successfully with a mean follow up time of 28 months. The patients included those with iatrogenic rectal injury during retropubic prostatectomy. Our case represents an injury due to high intensity focal ultrasound given for prostate cancer.

In a series of 11 cases by Zmora *et al* ^6^, 9 patients healed without complication and 2 others required further surgical management. Thus a success rate of 81% was achieved. This series included two patients with post radical prostatectomy fistula in two instances. The authors advocated this approach in failed previous repairs as in our case.

The technique of harvesting BMG was first described by Allen F. Morey *et al* ^7^ in 1996. Andrich DE ^8^ reported better results for dorsal as opposed to ventral onlay due to more vascular and better bed of corporal bodies for graft uptake. Further it was found that strictures in sittings of ischemia are better repaired with flaps due to poor surrounding blood supply. In our case the urethral defect was 2.5cm and in the prostatic urethra with local ischemia, thus a BMG without the grascilis muscle flap bed would result in a poorer outcome.

More recently Vanni *et al* ^9^ published case series of 74 patients with rectourethral fistula which included 2 patients with post- HIFU rectourethral fistula. There patients under went fistula repair with interposition muscle flaps with or without BMG with overall success rate of 84%. This article confirms the feasibility of combined BMG and gracilis muscle flap repair and thus provides a proof of concept for our case report.

## Conclusion

Rectourethral fistula secondary to HIFU should be categorised as acomplicated fistula owing to the hostile environment caused by the local heat generated by primary treatment. This report suggests rectourethral fistula post HIFU should be repaired with gracilis muscle flap with reinforced buccal mucosa graft as the “first chance in the best chance” in such situations.

## Key messages from our case report

1. Good exposure and adequate dissection is vital; this was achieved by the perineal approach.

2. Tension free repair of the rectum was achieved by dissection of rectum 2 cm cranial to the fistula and on the urethral side; buccal mucosa graft was used for tension free repair.

3. As both rectum and urethra are high pressure zones, there is a high probability of failure if both the repairs are not separated by live tissue ^10^. Ideal tissue for this interposition is a tissue with its own blood supply, in this case a pedicle gracilis muscle flap. The advantage of this flap was it acts as a physical barrier as well as a vascular bed for BMG.

4. Adequate haemostasis and good closure is equally important, as any collection is likely to get infected leading to recurrent fistula. Closure over suction drain helps in reducing the chances of collection, and also to keep buccal mucosal graft adherent to surrounding vascular tissue, thus helping in graft uptake.

## Consent

Written informed consent for publication of the patient’s clinical details and clinical images was obtained from the patient.
