# Supplementary material for: Case Report: Use of reinforced buccal mucosa graft over gracilis muscle flap in management of post high intensity focused ultrasound (HIFU) rectourethral fistula
Source: F1000Res. 2017 Feb 13;5:2891. Originally published 2016 Dec 21. [Version 2] doi: 10.12688/f1000research.10245.2 (PMC5310386; doi:10.12688/f1000research.10245.2)
Supplement: Peer review history

# Peer review: Referee response for version 2

**DOI:** 10.5256/f1000research.11661.r20161

**Published:** 2017-02-13

**Authors:** Alex J. Vanni

**Affiliations:** 1 Department of Urology, Institute of Urology, Lahey Hospital & Medical Center, Tufts University School of Medicine, Burlington, MA, USA

The authors have adequately addressed all concerns.

I have read this submission. I believe that I have an appropriate level of expertise to confirm that it is of an acceptable scientific standard.

# Peer review: Referee response for version 1

**DOI:** 10.5256/f1000research.11034.r18709

**Published:** 2017-01-09

**Authors:** Alex J. Vanni

**Affiliations:** 1 Department of Urology, Institute of Urology, Lahey Hospital & Medical Center, Tufts University School of Medicine, Burlington, MA, USA

This article demonstrates that a HIFU induced RUF can be successsfully closed with a buccal graft and gracilis muscle flap. I agree with the authors that this technique is the preferred way to treat these RUF.

 The article contends this is the first case demonstrating this in the literature. In fact, we published this first in 2010, in which 2 patients of our cohort had HIFU RUF that were successfully repaired with this technique. This report should be changed to appropriately acknowledge that we (Vanni *et al*) were the first to demonstrate closure of HIFU RUF with this technique with the appropriate reference cited.

 Here is this reference:  

Vanni AJ ^1^, Buckley JC, Zinman LN. Management of surgical and radiation induced rectourethral fistulas with an interposition muscle flap and selective buccal mucosal onlay graft. J Urol. 2010 Dec;184(6):2400-4.

 - Another point: In the discussion the authors state:

 "Zinman ^9 ^described 68 patients with rectourethral fistula who underwent gracilis muscle flap repair out of which 27 were performed in combination with BMG".

 This reference is antiquated and we have published 2 more extensive papers on the topic more recently that should be cited instead of the one used by the authors. Vanni *et al* is a better reference for this sentence and the one I previously mentioned above. In this paper, 74 patients had RUF repair with a gracilis muscle flap. 39 of these patients had a RUF from an ablative source (radiation and 2 HIFU). Of these 39 patients, 34 had a buccal mucosa graft (including the 2 HIFU cases) used to close the urethral defect. 37 of these patients had at least 1 gracilis muscle flap, while the other 2 patients had an inferior gluteus maximus flap and a Singapore fasciocutaneous flap.

I have read this submission. I believe that I have an appropriate level of expertise to confirm that it is of an acceptable scientific standard, however I have significant reservations, as outlined above.

# Peer review: Referee response for version 1

**DOI:** 10.5256/f1000research.11034.r18843

**Published:** 2016-12-29

**Authors:** Sanjay B. Kulkarni, Pankaj Joshi

**Affiliations:** 1 Kulkarni Reconstructive Urology Center, Pune, Maharashtra, India; 2 Department of Urology, Centre for Reconstructive Urology, Pune, Maharashtra, India

The authors have highlighted and important complex subject of post HIFU Recto Urethral fistula.

 As stated this should be classified as complex fistula.

 Management requires multiple options and an experienced team of Reconstructive Urologists.

 Using a vascularised flap is important interposition tissue.

 The authors have simultaneously augmented the urethra with buccal graft.

 We agree with all the key messages as highlighted by authors.

 We suggest this article be indexed and be made available as early as possible

We have read this submission. We believe that we have an appropriate level of expertise to confirm that it is of an acceptable scientific standard.
